# Supplementary material for: Spatiotemporal establishment of dense bacterial colonies growing on hard agar
Source: eLife. 2019 Mar 11;8:e41093. doi: 10.7554/eLife.41093 (PMC6411370; doi:10.7554/eLife.41093)
Supplement: Supplementary file 1. [file elife-41093-supp1.pdf]

Supplemental Tables

**Sugar Type**	**Batch Culture Growth Rate** (h^{-1})	**Initial Concentration** (mM)
Glucose	0.91	11.1
Arabinose	0.88	13.3
Mannitol	0.85	11.0
Maltose	0.84	5.80
Fructose	0.69	11.1
Melibiose	0.68	5.80
Sorbitol	0.61	11.0
Mannose	0.48	11.1

Table S1: A list of carbon sources with different batch culture growth rates and initial concentrations in the agar. Phosphate-buffered media (N-C-) was used for both batch and colony growth as described in Ref. [S2]. Various carbon sources were used as specified here. The concentration of all carbon sources used was 0.2% (w/v). Moreover, the molar carbon concentration for each carbon source was 66.7 mM. 10 mM NH_4Cl was added as the sole nitrogen source. To prepare the agar plate, we used agar with concentration 1.5% (w/v). 20 mL of molten agar gel was poured into 60 mm diameter dishes to a final thickness of approximately 7 mm, and allowed to cool at room temperature.

Symbol	Description	Value	Unit
K_{S}	nutrient capacity constant [a]	20	μM
Y	yield factor [b]	0.5	$g_{\mathrm{CDW}}/g_{\mathrm{glucose}}$
D_{-}	coefficient of nutrient diffusion in agar [c]	600	$\mu\mathrm{m}^2/\mathrm{s}$
D_{+}	coefficient of nutrient diffusion in colony [d]	90	$\mu\mathrm{m}^2/\mathrm{s}$
C_{s}	boundary value of nutrient concentration [e]	0.5	mM

Table S2: Parameters for nutrient.

[a] See Ref. [S3].

[b] See Ref. [S4].

[c] See Refs. [S7, 8].

[d] To estimate the diffusion coefficient D_{+} in the colony region, we use the formula $D_{+} = D_{-}(1-\phi)/\tau^2$ [S5], where ϕ is the volume fraction of cells and τ is the tortuosity. The volume fraction ϕ is defined to be the volume of all cells in a unit volume of the colony. It is found in our simulations that the closely packed cells have the volume fraction around $\phi = 0.7$ (cf. Fig. 3–figure supplement 1 in the main text), which is the value we take here. Capillary models yield $\tau = \sqrt{2}$ or $\tau = \sqrt{3}$ [S5]. Here we take $\tau = \sqrt{2}$ as an approximation. As a result, we have $D_{+} \approx 0.15 D_{-}$, which is $90\,\mu\mathrm{m}^2/\mathrm{s}$. Note that this value is within the range of an empirical estimation based on experimental data, which shows that for large solutes such as glucose in biofilm with 70% volume fraction, the ratio D_{+}/D_{-} is roughly 0.1–0.2 [S5].

[e] The value of C_{s} varies in our simulations. Here we only give a representative value.

Symbol	Description	Value	Unit
w_0	diameter of cell hemispherical caps [a]	1	μm
$\ell_{\rm div}$	cell dividing (cylindrical) length [b]	3	μm
ℓ_0	cylindrical length of a new born cell [c]	1	μm
$\ell_{\rm ran}$	max. length fluctuation in cell division	0.125	μm
$\varphi_{\rm ran}$	max. angle fluctuation in cell division	0.0005	rad
$\omega_{\rm ran}$	max. angular velocity fluctuation in cell division	0.0005	$\text{rad} \cdot \text{h}^{-1}$
$\lambda_{\rm S}$	batch culture growth rate [d]	1	h^{-1}
$\rho_{\rm cell}$	cell dry weight (CDW) of a typical mature cell [e]	0.146	$\text{pg} \cdot \mu\text{m}^{-3}$
ρ_0	constant cell density per unit volume of the colony [f]	0.102	$\text{pg} \cdot \mu\text{m}^{-3}$

Table S3: Parameters for cell geometry, and cell growth, division, and movement.

[a] The diameter w_0 of hemispherical caps of any cell is fixed during all of our simulations. We take it to be that of a mature *E. coli* cell.

[b] Based on experimental observations [S6], we set the value of $\ell_{\rm div}$ to be $3w_0$, which is a constant for all of our simulations. In general, the cell dividing length may depend on the batch culture growth rate $\lambda_{\rm S}$. The effect of our simplification with a constant dividing length is discussed in Appendix A2.2.

[c] The cylindrical length of any cell among those initially distributed on the agar surface in our simulations is also set to ℓ_0.

[d] This is also the maximum growth rate. It is a controllable parameter in our simulations. Here we list only a representative value.

[e] See Ref. [S1].

[f] The constant density ρ_0 is used in replace of a spatially varying local cell density (per unit colony volume) in updating the nutrient concentration filed; cf. Appendix A1.2. We have $\rho_0 = \phi_0 \rho_{\rm cell}$, where ϕ_0 is the constant volume fraction of cells per unit colony volume and is found to be around 0.68 in our simulations; cf. Appendix A2.1.

Symbol	Description	Value	Unit
μ_{cc}	cell-cell friction coefficient [a]	0.1	no unit
μ_{ca}	cell-agar friction coefficient [b]	0.8	no unit
k_{cc}	cell-cell Hertzian elastic constant [c]	30000	$\text{pg} \cdot \mu\text{m}^{-1} \cdot \text{h}^{-2}$
k_{ca}	cell-agar Hertzian elastic constant [d]	30000	$\text{pg} \cdot \mu\text{m}^{-1} \cdot \text{h}^{-2}$
$\gamma_{\text{cc},n}$	cell-cell normal dissipation rate [e]	10000	$\mu\text{m}^{-1} \cdot \text{h}^{-1}$
$\gamma_{\text{cc},t}$	cell-cell tangential dissipation rate [f]	100	$\mu\text{m}^{-1/2} \cdot \text{h}^{-1}$
$\gamma_{\text{ca},n}$	cell-agar normal dissipation rate [g]	10000	$\mu\text{m}^{-1} \cdot \text{h}^{-1}$
$\gamma_{\text{ca},t}$	cell-agar tangential dissipation rate [h]	100	$\mu\text{m}^{-1/2} \cdot \text{h}^{-1}$
γ_{surf}	surface tension constant [i]	150	$\text{pg} \cdot \text{h}^{-2}$
δh	tightness of water sticking to cells [j]	-0.01	μm
μ_{liq}	liquid viscosity [k]	0.03	$\text{pg} \cdot \mu\text{m}^{-1} \cdot \text{h}^{-1}$
h_{ran}	max. height fluctuation of agar surface	0.1	μm

Table S4: Parameters in force calculations. Little is known about the precise values of the many force parameters introduced in our model. Simulation stability demands that the largest time-step used in cellular simulations scales inversely with the largest force constant. In order to simulate long times for colony growth, we used force constants that are likely several orders of magnitude smaller than reality. Below is the rationale behind the parameters chosen.

[a,b] μ_{cc} and μ_{ca} have significant effects on colony morphology. The colony viscosity and the cell-agar friction are primarily determined by these parameters, as the viscous friction saturates rapidly.

[c,d] Values of k_{cc} and k_{ca} were chosen to be as small as possible while preventing unrealistically large cellular overlap. Results were insensitive to these parameters above this level, but increasing their values significantly slowed down simulations.

[e,f,g,h] The primary dissipation in our simulation is through cell-cell and cell-agar friction. Therefore, γ_{cc}, γ_{ca} were chosen to be large enough to damp most cellular vibrations. Results were insensitive to these parameters above what is required for stability.

[i] γ_{surf} is the surface tension parameter which sets the pressure scale within the colony.

[j] δh also causes small changes to the colony morphology. See Fig. 14 in the main text.

[k] μ_{liq} was chosen to be large enough to damp vibrations in the colony. This is primarily useful in the monolayer stage of colony growth. Results were not sensitive to this parameter.

[l] h_{ran} is useful in breaking the symmetry after division, preventing single lines of cells.

Symbol	Description	Value	unit
L	half side of the square base of the computational box [a]	3200	μm
a	depth of agar region from the mean agar surface [b]	960	μm
b	height of the regions of agar and air [c]	160	μm
N_x	number of grids in the x direction	200	no unit
N_y	number of grids in the y direction	200	no unit
N_{zc}	number of grids in the z direction in colony	40	no unit
N_{za}	number of grids in the z direction in agar	20	no unit
$N_{\mathrm{iter,min}}$	minimum iteration number for updating nutrient	10	no unit
$N_{\mathrm{iter,max}}$	maximum iteration number for updating nutrient	50000	no unit
h_{grid}	spatial grid size [d]	4	μm
err_{conv}	error tolerance in stopping criterion [e]	0.01	no unit
Δt	time step for cell growth [f]	0.00005	h

Table S5: Numerical parameters.

[a,b,c] See Fig. 11 in the main text for an illustration of the parameters L, a, and b.

[d] This is the grid size for the finest part of the multi-level grid; cf. Fig. A1.1 in Appendix A1.2. A total of 4 levels of grids were used in our simulations.

[e] This is a representative value.

[f] This is a representative value.

References

[S1] M. Basan, M. Zhu, X. Dai, M. Warren, D. Sévin, Y.-P. Wang, and T. Hwa. Inflating bacterial cells by increased protein expression. *Mol. Syst. Biol.*, 11(10), 2015.

[S2] L. N. Csonka, T. P. Ikeda, S. A. Fletcher, and S. Kustu. The accumulation of glutamate is necessary for optimal growth of *Salmonella typhimurium* in media of high osmolality but not induction of the proU operon. *J. Bacteriol.*, 176:6324–6333, 1994.

[S3] J. Monod. The growth of bacterial cultures. *Annu Rev Microbiol*, 3(1):371–394, 1949.

[S4] W. Payne. Energy yields and growth of heterotrophs. *Annu Rev Microbiol*, 24:17–52, 1970.

[S5] L. Shen and Z. Chen. Critical review of the impact of tortuosity on diffusion. *Chem. Eng. Sci.*, 62(14):3748–3755, 2007.

[S6] F. Si, D. Li, S. E. Cox, J. T. Sauls, O. Azizi, C. Sou, A. B. Schwartz, M. J. Erickstad, Y. Jun, X. Li, and S. Jun. Invariance of initiation mass and predictability of cell size in *Escherichia coli*. *Curr. Biol*, 27:1278–1287, 2017.

[S7] P. S. Stewart. A review of experimental measurements of effective diffusive permeabilities and effective diffusion coefficients in biofilms. *J. Bacteriology*, 59(3):262–272, 1998.

[S8] P. S. Stewart. Diffusion in biofilms. *J. Bacteriology*, 185(5):1845–1491, 2003.
